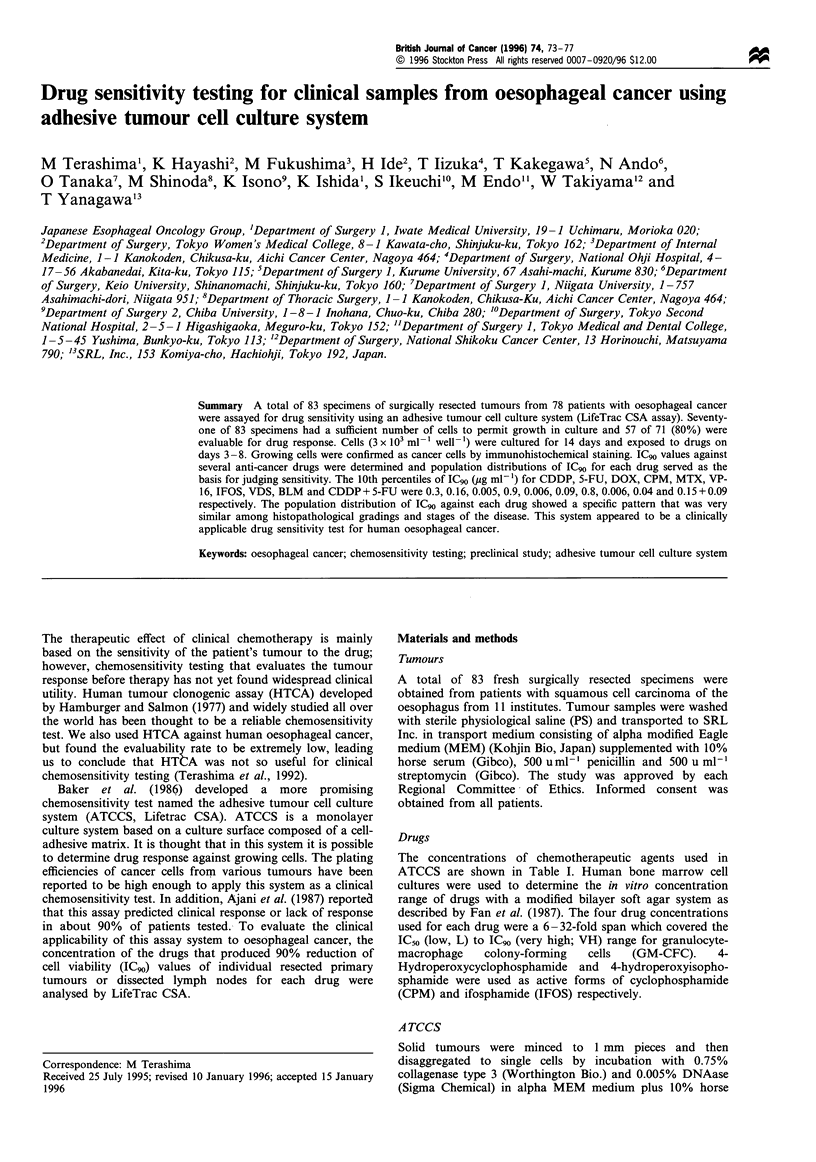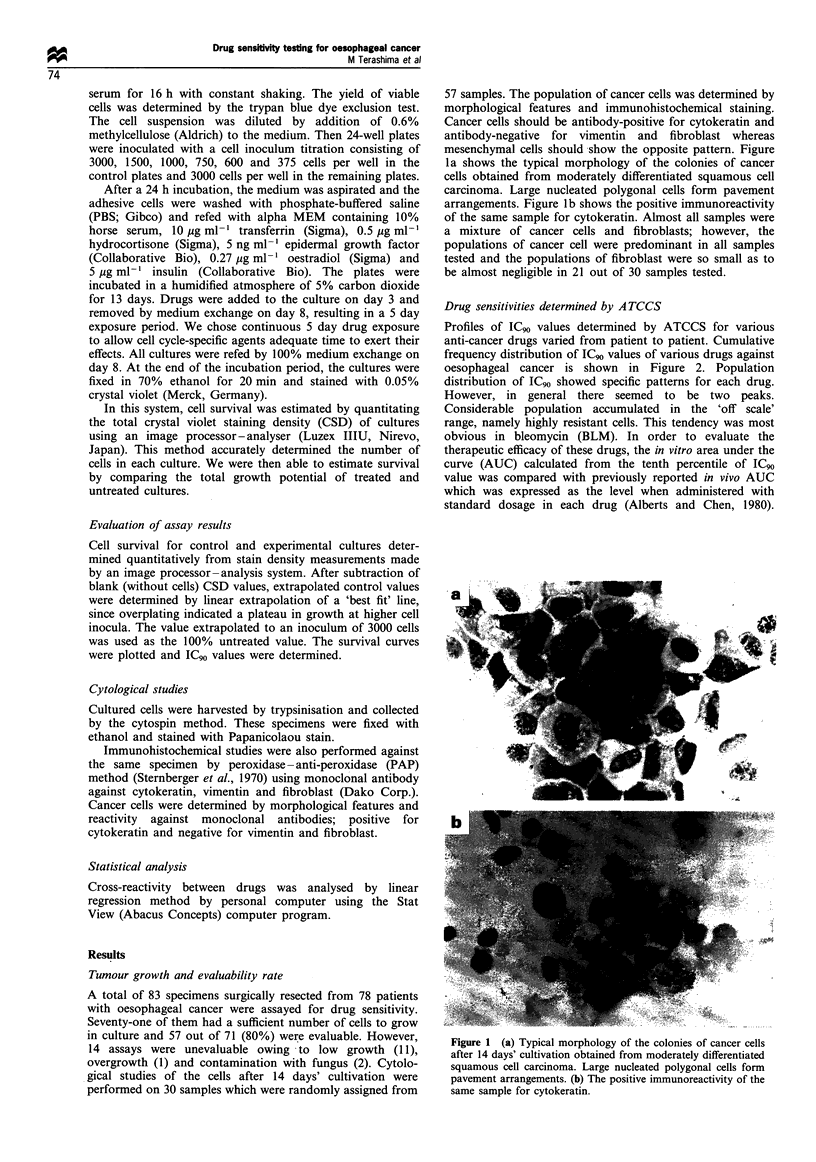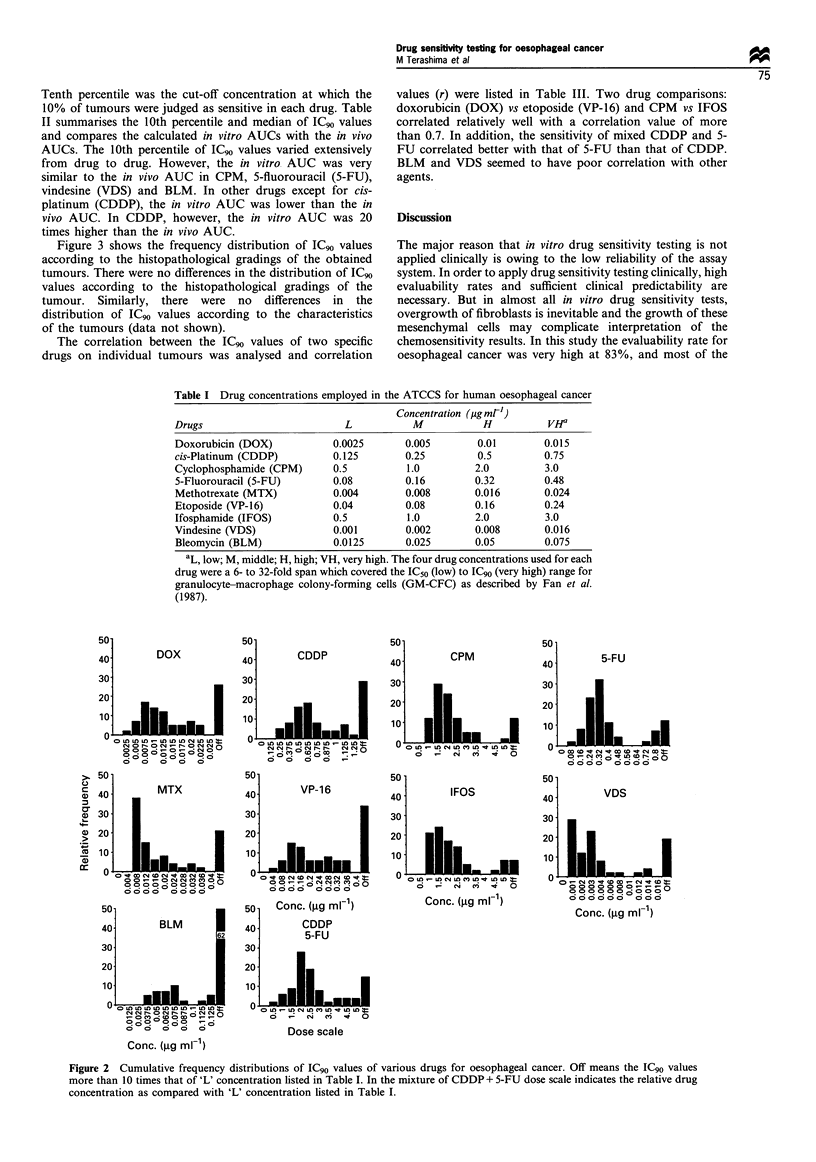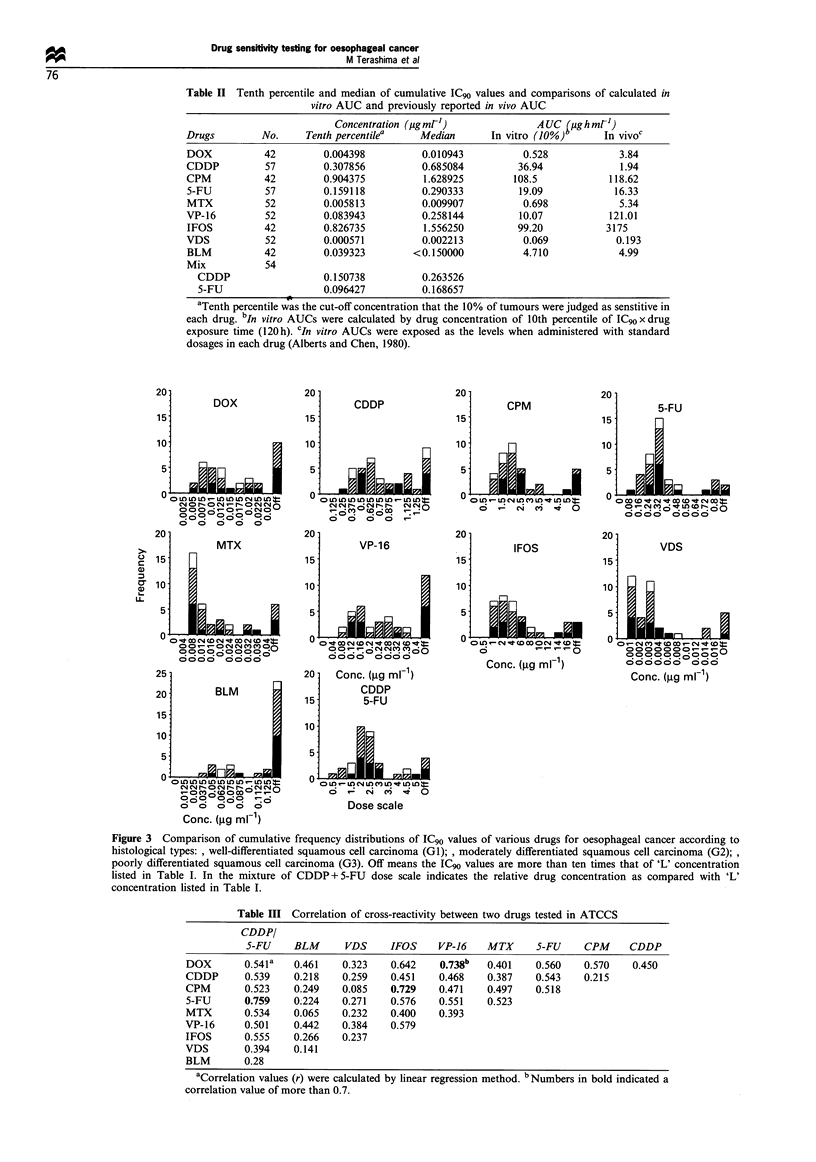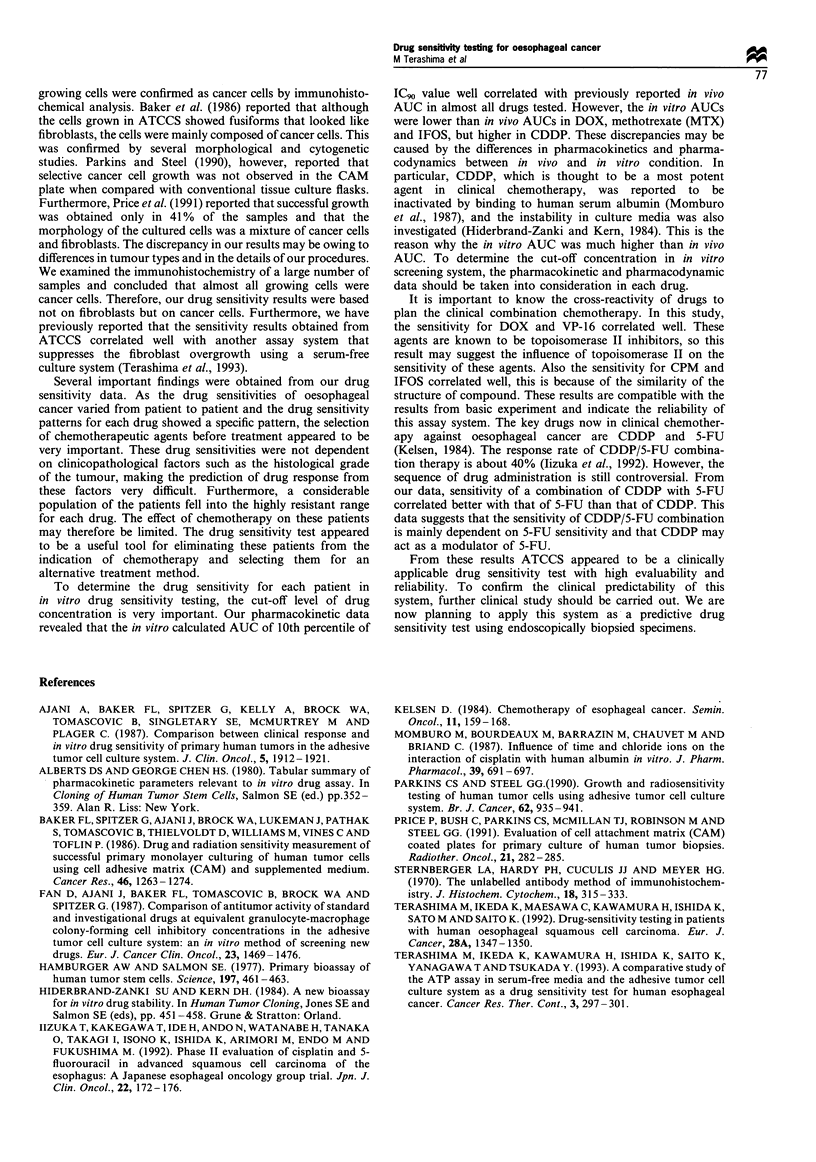# Drug sensitivity testing for clinical samples from oesophageal cancer using adhesive tumour cell culture system.

**DOI:** 10.1038/bjc.1996.318

**Published:** 1996-07

**Authors:** M. Terashima, K. Hayashi, M. Fukushima, H. Ide, T. Iizuka, T. Kakegawa, N. Ando, O. Tanaka, M. Shinoda, K. Isono, K. Ishida, S. Ikeuchi, M. Endo, W. Takiyama, T. Yanagawa

**Affiliations:** Department of Surgery, Iwate Medical University, Morioka, Japan.

## Abstract

**Images:**


British Journal of Cancer (1996) 74, 73-77
© 1996 Stockton Press All rights reserved 0007-0920/96 $12.00

### Drug sensitivity testing for clinical samples from oesophageal cancer using adhesive tumour cell culture system

M Terashima[1], K Hayashi[2], M Fukushima[3], H Ide[2], T Iizuka[4], T Kakegawa[5], N Ando[6], O Tanaka[7], M Shinoda[8], K Isono[9], K Ishida[1], S Ikeuchi[10], M Endo[11], W Takiyama[12] and T Yanagawa[13]

*Japanese Esophageal Oncology Group, [1]Department of Surgery 1, Iwate Medical University, 19-1 Uchimaru, Morioka 020; [2]Department of Surgery, Tokyo Women's Medical College, 8-1 Kawata-cho, Shinjuku-ku, Tokyo 162; [3]Department of Internal Medicine, 1-1 Kanokoden, Chikusa-ku, Aichi Cancer Center, Nagoya 464; [4]Department of Surgery, National Ohji Hospital, 4-17-56 Akabanedai, Kita-ku, Tokyo 115; [5]Department of Surgery 1, Kurume University, 67 Asahi-machi, Kurume 830; [6]Department of Surgery, Keio University, Shinanomachi, Shinjuku-ku, Tokyo 160; [7]Department of Surgery 1, Niigata University, 1-757 Asahimachi-dori, Niigata 951; [8]Department of Thoracic Surgery, 1-1 Kanokoden, Chikusa-Ku, Aichi Cancer Center, Nagoya 464; [9]Department of Surgery 2, Chiba University, 1-8-1 Inohana, Chuo-ku, Chiba 280; [10]Department of Surgery, Tokyo Second National Hospital, 2-5-1 Higashigaoka, Meguro-ku, Tokyo 152; [11]Department of Surgery 1, Tokyo Medical and Dental College, 1-5-45 Yushima, Bunkyo-ku, Tokyo 113; [12]Department of Surgery, National Shikoku Cancer Center, 13 Horinouchi, Matsuyama 790; [13]SRL, Inc., 153 Komiya-cho, Hachiohji, Tokyo 192, Japan.*

**Summary** A total of 83 specimens of surgically resected tumours from 78 patients with oesophageal cancer were assayed for drug sensitivity using an adhesive tumour cell culture system (LifeTrac CSA assay). Seventy-one of 83 specimens had a sufficient number of cells to permit growth in culture and 57 of 71 (80%) were evaluable for drug response. Cells ($3 \times 10^3$ ml$^{-1}$ well$^{-1}$) were cultured for 14 days and exposed to drugs on days 3-8. Growing cells were confirmed as cancer cells by immunohistochemical staining. $IC_{90}$ values against several anti-cancer drugs were determined and population distributions of $IC_{90}$ for each drug served as the basis for judging sensitivity. The 10th percentiles of $IC_{90}$ ($\mu$g ml$^{-1}$) for CDDP, 5-FU, DOX, CPM, MTX, VP-16, IFOS, VDS, BLM and CDDP+5-FU were 0.3, 0.16, 0.005, 0.9, 0.006, 0.09, 0.8, 0.006, 0.04 and 0.15+0.09 respectively. The population distribution of $IC_{90}$ against each drug showed a specific pattern that was very similar among histopathological gradings and stages of the disease. This system appeared to be a clinically applicable drug sensitivity test for human oesophageal cancer.

**Keywords:** oesophageal cancer; chemosensitivity testing; preclinical study; adhesive tumour cell culture system

The therapeutic effect of clinical chemotherapy is mainly based on the sensitivity of the patient's tumour to the drug; however, chemosensitivity testing that evaluates the tumour response before therapy has not yet found widespread clinical utility. Human tumour clonogenic assay (HTCA) developed by Hamburger and Salmon (1977) and widely studied all over the world has been thought to be a reliable chemosensitivity test. We also used HTCA against human oesophageal cancer, but found the evaluability rate to be extremely low, leading us to conclude that HTCA was not so useful for clinical chemosensitivity testing (Terashima *et al.*, 1992).

Baker *et al.* (1986) developed a more promising chemosensitivity test named the adhesive tumour cell culture system (ATCCS, Lifetrac CSA). ATCCS is a monolayer culture system based on a culture surface composed of a cell-adhesive matrix. It is thought that in this system it is possible to determine drug response against growing cells. The plating efficiencies of cancer cells from various tumours have been reported to be high enough to apply this system as a clinical chemosensitivity test. In addition, Ajani *et al.* (1987) reported that this assay predicted clinical response or lack of response in about 90% of patients tested. To evaluate the clinical applicability of this assay system to oesophageal cancer, the concentration of the drugs that produced 90% reduction of cell viability ($IC_{90}$) values of individual resected primary tumours or dissected lymph nodes for each drug were analysed by LifeTrac CSA.

Correspondence: M Terashima
Received 25 July 1995; revised 10 January 1996; accepted 15 January 1996

### Materials and methods

### *Tumours*

A total of 83 fresh surgically resected specimens were obtained from patients with squamous cell carcinoma of the oesophagus from 11 institutes. Tumour samples were washed with sterile physiological saline (PS) and transported to SRL Inc. in transport medium consisting of alpha modified Eagle medium (MEM) (Kohjin Bio, Japan) supplemented with 10% horse serum (Gibco), 500 u ml$^{-1}$ penicillin and 500 u ml$^{-1}$ streptomycin (Gibco). The study was approved by each Regional Committee of Ethics. Informed consent was obtained from all patients.

### *Drugs*

The concentrations of chemotherapeutic agents used in ATCCS are shown in Table I. Human bone marrow cell cultures were used to determine the *in vitro* concentration range of drugs with a modified bilayer soft agar system as described by Fan *et al.* (1987). The four drug concentrations used for each drug were a 6-32-fold span which covered the $IC_{50}$ (low, L) to $IC_{90}$ (very high; VH) range for granulocyte-macrophage colony-forming cells (GM-CFC). 4-Hydroperoxycyclophosphamide and 4-hydroperoxyisophosphamide were used as active forms of cyclophosphamide (CPM) and ifosphamide (IFOS) respectively.

### *ATCCS*

Solid tumours were minced to 1 mm pieces and then disaggregated to single cells by incubation with 0.75% collagenase type 3 (Worthington Bio.) and 0.005% DNAase (Sigma Chemical) in alpha MEM medium plus 10% horse serum for 16 h with constant shaking. The yield of viable cells was determined by the trypan blue dye exclusion test. The cell suspension was diluted by addition of 0.6% methylcellulose (Aldrich) to the medium. Then 24-well plates were inoculated with a cell inoculum titration consisting of 3000, 1500, 1000, 750, 600 and 375 cells per well in the control plates and 3000 cells per well in the remaining plates.

After a 24 h incubation, the medium was aspirated and the adhesive cells were washed with phosphate-buffered saline (PBS; Gibco) and refed with alpha MEM containing 10% horse serum, 10 $\mu g\ ml^{-1}$ transferrin (Sigma), 0.5 $\mu g\ ml^{-1}$ hydrocortisone (Sigma), 5 $ng\ ml^{-1}$ epidermal growth factor (Collaborative Bio), 0.27 $\mu g\ ml^{-1}$ oestradiol (Sigma) and 5 $\mu g\ ml^{-1}$ insulin (Collaborative Bio). The plates were incubated in a humidified atmosphere of 5% carbon dioxide for 13 days. Drugs were added to the culture on day 3 and removed by medium exchange on day 8, resulting in a 5 day exposure period. We chose continuous 5 day drug exposure to allow cell cycle-specific agents adequate time to exert their effects. All cultures were refed by 100% medium exchange on day 8. At the end of the incubation period, the cultures were fixed in 70% ethanol for 20 min and stained with 0.05% crystal violet (Merck, Germany).

In this system, cell survival was estimated by quantitating the total crystal violet staining density (CSD) of cultures using an image processor–analyser (Luzex IIIU, Nirevo, Japan). This method accurately determined the number of cells in each culture. We were then able to estimate survival by comparing the total growth potential of treated and untreated cultures.

### *Evaluation of assay results*

Cell survival for control and experimental cultures determined quantitatively from stain density measurements made by an image processor–analysis system. After subtraction of blank (without cells) CSD values, extrapolated control values were determined by linear extrapolation of a 'best fit' line, since overplating indicated a plateau in growth at higher cell inocula. The value extrapolated to an inoculum of 3000 cells was used as the 100% untreated value. The survival curves were plotted and $IC_{90}$ values were determined.

### *Cytological studies*

Cultured cells were harvested by trypsinisation and collected by the cytospin method. These specimens were fixed with ethanol and stained with Papanicolaou stain.

Immunohistochemical studies were also performed against the same specimen by peroxidase–anti-peroxidase (PAP) method (Sternberger *et al.*, 1970) using monoclonal antibody against cytokeratin, vimentin and fibroblast (Dako Corp.). Cancer cells were determined by morphological features and reactivity against monoclonal antibodies; positive for cytokeratin and negative for vimentin and fibroblast.

### *Statistical analysis*

Cross-reactivity between drugs was analysed by linear regression method by personal computer using the Stat View (Abacus Concepts) computer program.

### Results

### *Tumour growth and evaluability rate*

A total of 83 specimens surgically resected from 78 patients with oesophageal cancer were assayed for drug sensitivity. Seventy-one of them had a sufficient number of cells to grow in culture and 57 out of 71 (80%) were evaluable. However, 14 assays were unevaluable owing to low growth (11), overgrowth (1) and contamination with fungus (2). Cytological studies of the cells after 14 days' cultivation were performed on 30 samples which were randomly assigned from 57 samples. The population of cancer cells was determined by morphological features and immunohistochemical staining. Cancer cells should be antibody-positive for cytokeratin and antibody-negative for vimentin and fibroblast whereas mesenchymal cells should show the opposite pattern. Figure 1a shows the typical morphology of the colonies of cancer cells obtained from moderately differentiated squamous cell carcinoma. Large nucleated polygonal cells form pavement arrangements. Figure 1b shows the positive immunoreactivity of the same sample for cytokeratin. Almost all samples were a mixture of cancer cells and fibroblasts; however, the populations of cancer cell were predominant in all samples tested and the populations of fibroblast were so small as to be almost negligible in 21 out of 30 samples tested.

### *Drug sensitivities determined by ATCCS*

Profiles of $IC_{90}$ values determined by ATCCS for various anti-cancer drugs varied from patient to patient. Cumulative frequency distribution of $IC_{90}$ values of various drugs against oesophageal cancer is shown in Figure 2. Population distribution of $IC_{90}$ showed specific patterns for each drug. However, in general there seemed to be two peaks. Considerable population accumulated in the 'off scale' range, namely highly resistant cells. This tendency was most obvious in bleomycin (BLM). In order to evaluate the therapeutic efficacy of these drugs, the *in vitro* area under the curve (AUC) calculated from the tenth percentile of $IC_{90}$ value was compared with previously reported *in vivo* AUC which was expressed as the level when administered with standard dosage in each drug (Alberts and Chen, 1980).

**Figure 1** (**a**) Typical morphology of the colonies of cancer cells after 14 days' cultivation obtained from moderately differentiated squamous cell carcinoma. Large nucleated polygonal cells form pavement arrangements. (**b**) The positive immunoreactivity of the same sample for cytokeratin.

Tenth percentile was the cut-off concentration at which the 10% of tumours were judged as sensitive in each drug. Table II summarises the 10th percentile and median of $IC_{90}$ values and compares the calculated *in vitro* AUCs with the *in vivo* AUCs. The 10th percentile of $IC_{90}$ values varied extensively from drug to drug. However, the *in vitro* AUC was very similar to the *in vivo* AUC in CPM, 5-fluorouracil (5-FU), vindesine (VDS) and BLM. In other drugs except for *cis*-platinum (CDDP), the *in vitro* AUC was lower than the *in vivo* AUC. In CDDP, however, the *in vitro* AUC was 20 times higher than the *in vivo* AUC.

Figure 3 shows the frequency distribution of $IC_{90}$ values according to the histopathological gradings of the obtained tumours. There were no differences in the distribution of $IC_{90}$ values according to the histopathological gradings of the tumour. Similarly, there were no differences in the distribution of $IC_{90}$ values according to the characteristics of the tumours (data not shown).

The correlation between the $IC_{90}$ values of two specific drugs on individual tumours was analysed and correlation values (*r*) were listed in Table III. Two drug comparisons: doxorubicin (DOX) *vs* etoposide (VP-16) and CPM *vs* IFOS correlated relatively well with a correlation value of more than 0.7. In addition, the sensitivity of mixed CDDP and 5-FU correlated better with that of 5-FU than that of CDDP. BLM and VDS seemed to have poor correlation with other agents.

### Discussion

The major reason that *in vitro* drug sensitivity testing is not applied clinically is owing to the low reliability of the assay system. In order to apply drug sensitivity testing clinically, high evaluability rates and sufficient clinical predictability are necessary. But in almost all *in vitro* drug sensitivity tests, overgrowth of fibroblasts is inevitable and the growth of these mesenchymal cells may complicate interpretation of the chemosensitivity results. In this study the evaluability rate for oesophageal cancer was very high at 83%, and most of the

**Table I** Drug concentrations employed in the ATCCS for human oesophageal cancer

| Drugs | Concentration ($\mu g\,ml^{-1}$) L | M | H | VH[a] |
|---|---|---|---|---|
| Doxorubicin (DOX) | 0.0025 | 0.005 | 0.01 | 0.015 |
| *cis*-Platinum (CDDP) | 0.125 | 0.25 | 0.5 | 0.75 |
| Cyclophosphamide (CPM) | 0.5 | 1.0 | 2.0 | 3.0 |
| 5-Fluorouracil (5-FU) | 0.08 | 0.16 | 0.32 | 0.48 |
| Methotrexate (MTX) | 0.004 | 0.008 | 0.016 | 0.024 |
| Etoposide (VP-16) | 0.04 | 0.08 | 0.16 | 0.24 |
| Ifosphamide (IFOS) | 0.5 | 1.0 | 2.0 | 3.0 |
| Vindesine (VDS) | 0.001 | 0.002 | 0.008 | 0.016 |
| Bleomycin (BLM) | 0.0125 | 0.025 | 0.05 | 0.075 |

[a]L, low; M, middle; H, high; VH, very high. The four drug concentrations used for each drug were a 6- to 32-fold span which covered the $IC_{50}$ (low) to $IC_{90}$ (very high) range for granulocyte–macrophage colony-forming cells (GM-CFC) as described by Fan *et al.* (1987).

**Figure 2** Cumulative frequency distributions of $IC_{90}$ values of various drugs for oesophageal cancer. Off means the $IC_{90}$ values more than 10 times that of 'L' concentration listed in Table I. In the mixture of CDDP + 5-FU dose scale indicates the relative drug concentration as compared with 'L' concentration listed in Table I.

**Table II** Tenth percentile and median of cumulative $IC_{90}$ values and comparisons of calculated *in vitro* AUC and previously reported *in vivo* AUC

| Drugs | No. | Concentration ($\mu g\,ml^{-1}$) Tenth percentile[a] | Median | AUC ($\mu g\,h\,ml^{-1}$) In vitro (10%)[b] | In vivo[c] |
|---|---|---|---|---|---|
| DOX | 42 | 0.004398 | 0.010943 | 0.528 | 3.84 |
| CDDP | 57 | 0.307856 | 0.685084 | 36.94 | 1.94 |
| CPM | 42 | 0.904375 | 1.628925 | 108.5 | 118.62 |
| 5-FU | 57 | 0.159118 | 0.290333 | 19.09 | 16.33 |
| MTX | 52 | 0.005813 | 0.009907 | 0.698 | 5.34 |
| VP-16 | 52 | 0.083943 | 0.258144 | 10.07 | 121.01 |
| IFOS | 42 | 0.826735 | 1.556250 | 99.20 | 3175 |
| VDS | 52 | 0.000571 | 0.002213 | 0.069 | 0.193 |
| BLM | 42 | 0.039323 | <0.150000 | 4.710 | 4.99 |
| Mix | 54 | | | | |
| CDDP | | 0.150738 | 0.263526 | | |
| 5-FU | | 0.096427 | 0.168657 | | |

[a]Tenth percentile was the cut-off concentration that the 10% of tumours were judged as senstitive in each drug. [b]*In vitro* AUCs were calculated by drug concentration of 10th percentile of $IC_{90} \times$ drug exposure time (120 h). [c]*In vitro* AUCs were exposed as the levels when administered with standard dosages in each drug (Alberts and Chen, 1980).

**Figure 3** Comparison of cumulative frequency distributions of $IC_{90}$ values of various drugs for oesophageal cancer according to histological types: , well-differentiated squamous cell carcinoma (G1); , moderately differentiated squamous cell carcinoma (G2); , poorly differentiated squamous cell carcinoma (G3). Off means the $IC_{90}$ values are more than ten times that of 'L' concentration listed in Table I. In the mixture of CDDP + 5-FU dose scale indicates the relative drug concentration as compared with 'L' concentration listed in Table I.

**Table III** Correlation of cross-reactivity between two drugs tested in ATCCS

| | CDDP/ 5-FU | BLM | VDS | IFOS | VP-16 | MTX | 5-FU | CPM | CDDP |
|---|---|---|---|---|---|---|---|---|---|
| DOX | 0.541[a] | 0.461 | 0.323 | 0.642 | **0.738**[b] | 0.401 | 0.560 | 0.570 | 0.450 |
| CDDP | 0.539 | 0.218 | 0.259 | 0.451 | 0.468 | 0.387 | 0.543 | 0.215 | |
| CPM | 0.523 | 0.249 | 0.085 | **0.729** | 0.471 | 0.497 | 0.518 | | |
| 5-FU | **0.759** | 0.224 | 0.271 | 0.576 | 0.551 | 0.523 | | | |
| MTX | 0.534 | 0.065 | 0.232 | 0.400 | 0.393 | | | | |
| VP-16 | 0.501 | 0.442 | 0.384 | 0.579 | | | | | |
| IFOS | 0.555 | 0.266 | 0.237 | | | | | | |
| VDS | 0.394 | 0.141 | | | | | | | |
| BLM | 0.28 | | | | | | | | |

[a]Correlation values ($r$) were calculated by linear regression method. [b]Numbers in bold indicated a correlation value of more than 0.7.

growing cells were confirmed as cancer cells by immunohistochemical analysis. Baker *et al.* (1986) reported that although the cells grown in ATCCS showed fusiforms that looked like fibroblasts, the cells were mainly composed of cancer cells. This was confirmed by several morphological and cytogenetic studies. Parkins and Steel (1990), however, reported that selective cancer cell growth was not observed in the CAM plate when compared with conventional tissue culture flasks. Furthermore, Price *et al.* (1991) reported that successful growth was obtained only in 41% of the samples and that the morphology of the cultured cells was a mixture of cancer cells and fibroblasts. The discrepancy in our results may be owing to differences in tumour types and in the details of our procedures. We examined the immunohistochemistry of a large number of samples and concluded that almost all growing cells were cancer cells. Therefore, our drug sensitivity results were based not on fibroblasts but on cancer cells. Furthermore, we have previously reported that the sensitivity results obtained from ATCCS correlated well with another assay system that suppresses the fibroblast overgrowth using a serum-free culture system (Terashima *et al.*, 1993).

Several important findings were obtained from our drug sensitivity data. As the drug sensitivities of oesophageal cancer varied from patient to patient and the drug sensitivity patterns for each drug showed a specific pattern, the selection of chemotherapeutic agents before treatment appeared to be very important. These drug sensitivities were not dependent on clinicopathological factors such as the histological grade of the tumour, making the prediction of drug response from these factors very difficult. Furthermore, a considerable population of the patients fell into the highly resistant range for each drug. The effect of chemotherapy on these patients may therefore be limited. The drug sensitivity test appeared to be a useful tool for eliminating these patients from the indication of chemotherapy and selecting them for an alternative treatment method.

To determine the drug sensitivity for each patient in *in vitro* drug sensitivity testing, the cut-off level of drug concentration is very important. Our pharmacokinetic data revealed that the *in vitro* calculated AUC of 10th percentile of $IC_{90}$ value well correlated with previously reported *in vivo* AUC in almost all drugs tested. However, the *in vitro* AUCs were lower than *in vivo* AUCs in DOX, methotrexate (MTX) and IFOS, but higher in CDDP. These discrepancies may be caused by the differences in pharmacokinetics and pharmacodynamics between *in vivo* and *in vitro* condition. In particular, CDDP, which is thought to be a most potent agent in clinical chemotherapy, was reported to be inactivated by binding to human serum albumin (Momburo *et al.*, 1987), and the instability in culture media was also investigated (Hiderbrand-Zanki and Kern, 1984). This is the reason why the *in vitro* AUC was much higher than *in vivo* AUC. To determine the cut-off concentration in *in vitro* screening system, the pharmacokinetic and pharmacodynamic data should be taken into consideration in each drug.

It is important to know the cross-reactivity of drugs to plan the clinical combination chemotherapy. In this study, the sensitivity for DOX and VP-16 correlated well. These agents are known to be topoisomerase II inhibitors, so this result may suggest the influence of topoisomerase II on the sensitivity of these agents. Also the sensitivity for CPM and IFOS correlated well, this is because of the similarity of the structure of compound. These results are compatible with the results from basic experiment and indicate the reliability of this assay system. The key drugs now in clinical chemotherapy against oesophageal cancer are CDDP and 5-FU (Kelsen, 1984). The response rate of CDDP/5-FU combination therapy is about 40% (Iizuka *et al.*, 1992). However, the sequence of drug administration is still controversial. From our data, sensitivity of a combination of CDDP with 5-FU correlated better with that of 5-FU than that of CDDP. This data suggests that the sensitivity of CDDP/5-FU combination is mainly dependent on 5-FU sensitivity and that CDDP may act as a modulator of 5-FU.

From these results ATCCS appeared to be a clinically applicable drug sensitivity test with high evaluability and reliability. To confirm the clinical predictability of this system, further clinical study should be carried out. We are now planning to apply this system as a predictive drug sensitivity test using endoscopically biopsied specimens.

### References

AJANI A, BAKER FL, SPITZER G, KELLY A, BROCK WA, TOMASCOVIC B, SINGLETARY SE, MCMURTREY M AND PLAGER C. (1987). Comparison between clinical response and *in vitro* drug sensitivity of primary human tumors in the adhesive tumor cell culture system. *J. Clin. Oncol.*, **5**, 1912–1921.

ALBERTS DS AND GEORGE CHEN HS. (1980). Tabular summary of pharmacokinetic parameters relevant to *in vitro* drug assay. In *Cloning of Human Tumor Stem Cells*, Salmon SE (ed.) pp.352–359. Alan R. Liss: New York.

BAKER FL, SPITZER G, AJANI J, BROCK WA, LUKEMAN J, PATHAK S, TOMASCOVIC B, THIELVOLDT D, WILLIAMS M, VINES C AND TOFLIN P. (1986). Drug and radiation sensitivity measurement of successful primary monolayer culturing of human tumor cells using cell adhesive matrix (CAM) and supplemented medium. *Cancer Res.*, **46**, 1263–1274.

FAN D, AJANI J, BAKER FL, TOMASCOVIC B, BROCK WA AND SPITZER G. (1987). Comparison of antitumor activity of standard and investigational drugs at equivalent granulocyte-macrophage colony-forming cell inhibitory concentrations in the adhesive tumor cell culture system: an *in vitro* method of screening new drugs. *Eur. J. Cancer Clin. Oncol.*, **23**, 1469–1476.

HAMBURGER AW AND SALMON SE. (1977). Primary bioassay of human tumor stem cells. *Science*, **197**, 461–463.

HIDERBRAND-ZANKI SU AND KERN DH. (1984). A new bioassay for *in vitro* drug stability. In *Human Tumor Cloning*, Jones SE and Salmon SE (eds), pp. 451–458. Grune & Stratton: Orland.

IIZUKA T, KAKEGAWA T, IDE H, ANDO N, WATANABE H, TANAKA O, TAKAGI I, ISONO K, ISHIDA K, ARIMORI M, ENDO M AND FUKUSHIMA M. (1992). Phase II evaluation of cisplatin and 5-fluorouracil in advanced squamous cell carcinoma of the esophagus: A Japanese esophageal oncology group trial. *Jpn. J. Clin. Oncol.*, **22**, 172–176.

KELSEN D. (1984). Chemotherapy of esophageal cancer. *Semin. Oncol.*, **11**, 159–168.

MOMBURO M, BOURDEAUX M, BARRAZIN M, CHAUVET M AND BRIAND C. (1987). Influence of time and chloride ions on the interaction of cisplatin with human albumin *in vitro*. *J. Pharm. Pharmacol.*, **39**, 691–697.

PARKINS CS AND STEEL GG.(1990). Growth and radiosensitivity testing of human tumor cells using adhesive tumor cell culture system. *Br. J. Cancer*, **62**, 935–941.

PRICE P, BUSH C, PARKINS CS, MCMILLAN TJ, ROBINSON M AND STEEL GG. (1991). Evaluation of cell attachment matrix (CAM) coated plates for primary culture of human tumor biopsies. *Radiother. Oncol.*, **21**, 282–285.

STERNBERGER LA, HARDY PH, CUCULIS JJ AND MEYER HG. (1970). The unlabelled antibody method of immunohistochemistry. *J. Histochem. Cytochem.*, **18**, 315–333.

TERASHIMA M, IKEDA K, MAESAWA C, KAWAMURA H, ISHIDA K, SATO M AND SAITO K. (1992). Drug-sensitivity testing in patients with human oesophageal squamous cell carcinoma. *Eur. J. Cancer*, **28A**, 1347–1350.

TERASHIMA M, IKEDA K, KAWAMURA H, ISHIDA K, SAITO K, YANAGAWA T AND TSUKADA Y. (1993). A comparative study of the ATP assay in serum-free media and the adhesive tumor cell culture system as a drug sensitivity test for human esophageal cancer. *Cancer Res. Ther. Cont.*, **3**, 297–301.